# Information Flow and Data Gaps in COVID-19 Recording and Reporting at National and Provincial Levels in Indonesia

**DOI:** 10.3390/healthcare10020204

**Published:** 2022-01-20

**Authors:** Diana Barsasella, Arief Tarmansyah Iman, Fery Fadly, Mohy Uddin, Arshad Mohammed, Tazeem Shaik, Hermawan Saputra, Shwetambara Malwade, Eshita Dhar, Zakiah, Nurhadi, Jitendra Jonnagaddala, Shabbir Syed-Abdul

**Affiliations:** 1 Graduate Institute of Biomedical Informatics, College of Medical Science and Technology, Taipei Medical University, Taipei 106, Taiwan; diana.barsasella5@gmail.com (D.B.); eshitadhar7@gmail.com (E.D.); 2 International Center for Health Information Technology (ICHIT), College of Medical Science and Technology, Taipei Medical University, Taipei 106, Taiwan; sv14.kekade@gmail.com; 3 Department of Medical Record and Health Information, Health Polytechnic of the Ministry of Health Tasikmalaya, Tasikmalaya 46115, West Java, Indonesia; arieftarmansyah@gmail.com (A.T.I.); fery.fadly@dosen.poltekkestasikmalaya.ac.id (F.F.); 4 Research Quality Management Section, King Abdullah International Medical Research Center, King Saud bin Abdulaziz University for Health Sciences, Ministry of National Guard-Health Affairs, Riyadh 11481, Saudi Arabia; drmohyuddin@yahoo.com; 5 Northside Clinics, Hyderabad 500082, India; arshadhere@gmail.com (A.M.); tazeemshaik@gmail.com (T.S.); 6 Department of Postgraduate, University of Muhammadiyah Prof Dr Hamka, Jakarta 12130, Indonesia; Hermawan.saputradr@gmail.com; 7 Health Department of Depok City, Depok 16431, West Java, Indonesia; akuinizakiah@gmail.com (Z.); hadi4us@gmail.com (N.); 8 School of Public Health and Community Medicine, University of New South Wales, Kensington, NSW 2033, Australia; 9 School of Gerontology Health Management, College of Nursing, Taipei Medical University, Taipei 106, Taiwan

**Keywords:** healthcare system, COVID-19 reporting, health surveillance, data collection, information flow

## Abstract

Epidemiological surveillance is an essential component of public health practice especially during infectious disease outbreaks. It is critical to offer transparent epidemiological information in a rigorous manner at different regional levels in countries for managing the outbreak situations. The objectives of this research are to better understand the information flow of COVID-19 health monitoring systems and to determine the data gaps of COVID-19 incidence at the national and provincial levels in Indonesia. COVID-19 information flow was researched using government websites at the national and various provincial levels. To find the disparities, we assessed the number of cases reported at both levels at the same time and displayed the absolute and relative differences. The findings revealed that out of a total of 34 provinces in Indonesia, data differences were seen in 25 (73.52%) provinces in terms of positive cases, 31 (91.18%) provinces in terms of cured cases, and 28 (82.35%) provinces of the number of deaths. Our results showed a pressing need for high-quality, transparent, and timely information. The integration of COVID-19 data in Indonesia has not been optimal, implying that the reported COVID-19 incidence rate may be biased or delayed. COVID-19 incidents must be better monitored to disrupt the disease’s transmission chain.

## 1. Introduction

In December 2019, the Severe Acute Respiratory Syndrome Coronavirus 2 (SARS-CoV-2) developed in Wuhan, Hubei Region, China, as a progressive episode of pneumonia linked to a novel coronavirus illness (COVID-19). The epidemic quickly spread throughout China and other countries around the world in the months that followed [1]. In situations like this, the importance of both traditional and social media cannot be overstated since proper distribution of epidemic information to the public is critical. The media’s channels and technologies can play a vital role in the distribution and exchange of information, scientific results, therapies, and protocols by crossing all geographical barriers. On the other hand, in addition to the benefits listed above, there are some drawbacks, such as the possibility that the information is erroneous or incorrect. This type of misinformation can perpetuate falsehoods and conspiracies, putting people’s health, safety, and the environment in jeopardy. Rapid and precise information transmission, case detection, data sharing, fluid communication, and peer-reviewed analysis are all critical in this time of uncertainty [2]. In epidemic outbreaks, it is critical to create accurate, high-quality, solid evidence that can aid the establishment of reliable information for public health decision-making [3].

Data collection, processing, analysis, and distribution are used in health surveillance to produce measurable information. Evidence-based measures require accurate, valid, and trustworthy epidemiological data, which may be used as the foundation for decision-making by competent authorities [4]. The development of an information system capable of studying confirmed, cured, and deceased patients is critical, especially when these systems can give real-time data. Complete and timely information is required for a responsive and successful COVID-19 surveillance system, and it must be compatible with its constituent clinical and public health information systems [5]. To aid the government in making choices, real-time metric data is needed to track the spread of COVID-19, such as new cases per day, cured patients per day, and deaths per day [6]. It can help the government to improve the management and make decisions for curbing the spread of COVID-19 according to the data reported in each province.

Epidemiological surveillance is a critical component of public health practice, as it allows for the monitoring of disease dissemination, the identification of disease progression trends, and the implementation of disease prevention and control strategies [7]. Different systems/tools have been established at the worldwide level that use epidemiological data to thoroughly monitor and track COVID-19 cases internationally, according to the literature [7]. The Centre for Systems Science and Engineering (CSSE) at Johns Hopkins University built a dashboard that can display data on instances that occur around the world and gives information on the incidence of COVID-19 cases worldwide using data sources such as Chinese CDC and WHO reports [8]. This system is updated twice a day to verify the accuracy of the data. Arneson et al. constructed an interactive website in the same way that outlines the investigation of coronavirus outbreaks [9]. The data is updated daily, and data quality is ensured through manual verification. An integrative review was conducted utilizing multiple web sources such as WHO, Euro-Surveillance, CDC, MOH, Medline, and PubMed, among others [7]. It emphasized key indicators such as accurate and timely surveillance data, challenges such as resource and training concerns affecting surveillance quality, and limits such as incomplete/lack of timeliness and completeness for surveillance data and reporting.

Since Indonesia had the highest number of fatalities in South East Asia and the lowest global screening rate, the government’s first response to the pandemic was to form a COVID-19 response acceleration task force, which was overseen by the National Disaster Management Agency [10]. Despite the tactics and control mechanisms in place, the number of suspected cases, confirmed cases, and deaths due to the pandemic were on the rise as of early 2021 [11]. To predict the outbreak of the infection, it is essential to efficiently track the epidemiological information in a robust manner at different provincial levels. The information needs to be accurate, reliable and should also be accessible to the public to follow the preventive measures in particular regions. The goal of this research is to better understand the information flow of COVID-19 health monitoring systems at different levels of the Indonesian government. It also aims to identify weaknesses at the provincial level and make recommendations for how to develop a comprehensive system at the national level to lessen the pandemic’s impact.

## 2. Materials and Methods

The information flow of COVID-19 was identified and studied in this study utilizing the following resources: the government website [11] at the national level, various provincial websites for provincial levels, and the Minister of Health of the Republic of Indonesia’s decree [12]. KawalCOVID-19 and Kawal Corona are two national websites [13,14] that were used to compile statistics on COVID-19 cases across Indonesia. The regulation of Permenkes Nomor 45 Tahun 2014 governs the surveillance of COVID-19 data on certain websites [15]. This entails collecting, reporting, and processing data, as well as disseminating information based on national needs in order to develop COVID-19 preventive and control policies. The order Hk.01.07/Menkes/413/2020 established guidelines for COVID-19 prevention, control, reporting, and monitoring [16].

We tracked and compared data from these websites at the national and provincial levels, covering all of Indonesia’s provinces at the same time, including Jakarta, Bogor, West Java, Central Java, and East Java. We also used these sources to calculate the number of affected cases, cured cases, and deaths at the national level. We looked at the disparities in case counts between data collected at the national level (through government websites) and data collected at the provincial level (through local government websites). A team of five members reviewed the data from the websites. Each member visited the national and provincial sites of each province. A total of seven days was taken to view, note, analyze, and find the gaps between national and provincial sites’ data. Each of the team members retrieved the same findings with regards to differences in the incidence of COVID-19 data.

## 3. Results

### 3.1. Information Flow of COVID-19 Recording and Reporting

**Figure 1:**
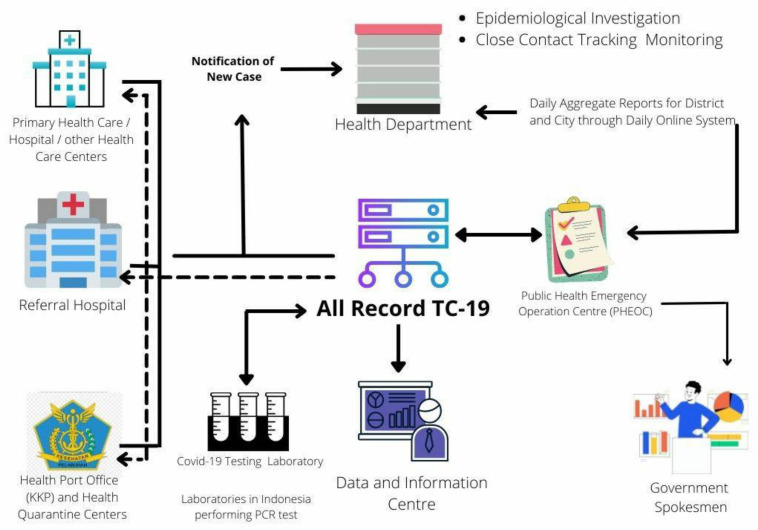
Information flow of COVID-19 recording and reporting [16].

The information flow (see Figure 1) [16] depicts the steps of information processing for COVID-19 reporting and recording from the provincial to the national level and demonstrates how Indonesia’s healthcare system operates. To identify data gaps and plausible reasons for these gaps for COVID-19 surveillance at the provincial and national levels in Indonesia, a thorough understanding of information flow is necessary. The information flow serves as proof of a thorough examination of the data flow throughout the system, as well as the identification of gaps and the likely causes of these gaps. It also aids in determining when the gaps may have occurred, making approach suggestions easier to resolve.

All confirmed COVID-19 cases that undergo PCR tests at various health facilities and hospitals, such as Primary Healthcare Centres, Referral Hospitals, Port Health Offices, and Health Quarantine Centres, as well as COVID-19 test laboratories, are reported at the provincial level to the national level database, All-Record TC-19 [17]. In the event of any discrepancies, the data can be forwarded back to provincial health facilities for validation. Furthermore, the data from the All-Record TC-19 is transmitted to the provincial Health Department for validation. At this stage, epidemiological research, as well as close contact tracing and monitoring, are carried out. The instances are also supported by daily reports that are compiled in aggregate. The data is then forwarded to the Indonesian Ministry of Health’s Public Health Emergency Operating Centre (PHEOC), which maintains epidemiological data and is part of the Indonesian Ministry of Health. It is then returned to the All-Record TC-19 database for final confirmation, after which the data is placed into the final records and forwarded toward news briefing and public publication.

#### 3.1.1. Case Finding and Epidemiological Investigation Report

Input to the Sistem Informasi Rumah Sakit (SIRS) Online program (Directorate General of Health Service—Ministry of Health, Jakarta, Indonesia) is provided by case data from registered hospitals in an online system called the Hospital Information System (SIRS) based on the provision of hospital services. They must enter the information into the All-Record TC-19 web program for health facilities that collect specimens after recording it. For recapitulation and follow-up, the case finding notification form is delivered to the District/City Health Office and the Provincial Health Office. Case data must be entered into the All-Record TC-19 program by healthcare facilities (Fasyankes) that provide case care or case monitoring (independent isolation) [17]. If the health facility is registered in the SIRS-Online application, then they ensure that both applications have the same data filled in. If they cannot report online, the health facility must complete the attached epidemiology investigation (Penyelidikan Epidemiologi) form and send it to the District/City Health Office for completion and further follow-up.

#### 3.1.2. Aggregate Daily Reports

The COVID-19 Daily Reporting Online System application is used by the District/City Health Office to generate the aggregate daily reports [17]. Case finding notification reports, specimen-related reports, PE reports, and close contact monitoring reports are all summarized in the daily report. The daily report is also used by the Health Office Regencies/Cities to keep track of case progress in their respective regions. Surveillance performance metrics, such as report completeness and correctness, are used in the daily reporting system. The report’s completeness indicates the number of districts/cities reporting in each province per day. Meanwhile, the report’s accuracy is constrained by the time it is taken each day, which is 12.00 noon Western Indonesian Time (WIB). The report’s accuracy demonstrates that the data was ready for policyholders to use on that day.

#### 3.1.3. Processing and Analysis of Data

The validator (Walidata) grants access permissions to the data received by the All-Record TC-19 application and the data processing unit to carry out the analysis according to the government’s needs. This is carried out at nearly all levels, including health facilities, laboratories, health departments, the Health Port Authority (KKP), and the Ministry of Health, as well as other relevant and deprived sectors.

#### 3.1.4. Data and Information Distribution

One Health Data Dashboard Application [17], which is publicly available, can access data obtained through the All-Record TC-19 system. The system’s data can be accessed directly by associated units, such as health facilities, laboratories, and health offices. Individual data can be accessed by the Ministry of Health and health offices both during and after the outbreak.

### 3.2. Comparison of Monitoring COVID-19 Incidence Data at Provincial and National Levels

We reviewed data from both national and provincial government websites to see if there were any differences in the incidence of COVID-19 (see Table 1).

For the province of Gorontalo, positive case and cured case figures were quite different between the sites at the national and provincial level. There was a significant variation in data between the data collected by the provincial administration of South Sulawesi and the data collected at the national level. The remaining 24 provinces had the incidence differences of positive cases, cured cases, and death. The biggest difference in terms of positive cases between the national and provincial levels was from Banten province (2824 cases). With regards to the number of cured cases, Papua province had the largest deviation (10,478 instances) from the national average. Central Java had the largest disparity in mortality between national and provincial levels, with the highest number of cases (5251 cases). On the other hand, there were a few provinces that had no change in terms of positive cases, cured cases, and deaths (0, 0, 0) when compared to national numbers, including West Java and South Kalimantan.

On the website of Bangka Belitung Province, we simply found an empty page [48]. Bangka, Bangka Barat, Bangka Tengah, Bangka Selatan, Pangkalpinang, Belitung, and Belitung Timur are the districts that make up the province of Bangka Belitung. We looked at the websites of the districts [52,53,54,55,56,57,58] and attempted to count the total number of cases in the province of Bangka Belitung, however, we discovered outdated data in two districts. The district of Bangka Selatan’s COVID-19 reported the cases data was last updated on 27 July 2020, while Pangkalpinang’s data was last updated on 9 August 2020. This condition implies that there are more inconsistencies based on data reported in Table 1.

## 4. Discussion

### 4.1. Finding of This Study

The information flow of COVID-19 from the province level to the national level in Indonesia was recorded and studied in our study. Subsequently, we discovered several conflict situations in some areas. There was a significant variation in the incidence of cured cases and death data between the data collected by the provincial administration and the national level in DKI Jakarta, Central Java, East Java, and Maluku. In the same way, the province of Bali and North Sumatra also experienced a similar scenario where the incidence of cured cases for the national and the provincial levels was different. Significant disparities were detected in the COVID-19 data at the provincial and national levels. Out of a total of 34 provinces in Indonesia, data differences were seen in 25 (73.52%) provinces in terms of positive cases, in 31 (91.18%) provinces in terms of cured cases, and 28 (82.35%) provinces for the number of deaths. These discrepancies hint at anomalies in health information systems for monitoring COVID-19 data, highlighting the necessity for adequate COVID-19 monitoring system integration at the provincial and national levels.

The data at the national level is derived from daily data acquired from PCR test results supplied to All Record TC-19 by hospitals and designated laboratories. A PCR test was applied only after the result of a rapid antibody test was positive and for those with close contact positive cases. Thus, a rapid antibody test was only performed for screening purposes [16]. Rapid antigen testing was applied and reported only later in 2021. There are 830 COVID-19 testing laboratories in Indonesia [59]. According to a report from the National Agency for Disaster Management (BNPB), only 52% of laboratories in Indonesia performed and reported PCR tests [60], to the provincial level agency and to All Record TC-19. This could be the initial cause of data disparities across provinces and at the national level. Due to a lack of suitable reporting resources, the remaining 48% of laboratories simply reported test findings at the provincial level. This indicates the need for PCR tests to be introduced in all 830 testing laboratories in the country to ensure efficient data reporting. Another cause for the data discrepancies could be the delays or various time periods for submitting data from the provincial to the national level [61]. According to a news report, data input delays resulted in 2 million COVID-19 testing results being under-reported in February 2021, potentially affecting the COVID-19 positivity rate in Indonesia [62]. Determining a specific time period of the day for data reporting from all the provinces could possibly aid in avoiding the data input delays.

COVID-19 data collection at the provincial level takes time since it must be reported to both the provincial and national levels at the same time. Even though the systems at the national and provincial levels are separate, the persons who enter the data are the same and require more time due to the large number of instances. This may have resulted in temporary discrepancies in the data at two levels [63]. Only a few provinces, including Jawa Barat and Kalimantan Selatan, showed no changes in the number of cases recorded at both levels. At such times, incidents of data dualism at the provincial level were seen, involving the separation of data into two types: data for publication in the media and real-time data. Depok City, for example, in West Java Province, utilized real-time data despite a significant disparity with national data [64]. As a result, some provinces may release data for publishing that is identical to national statistics, even though the actual figures in real-time data may differ. Their refusal to release real-time data could be due to their relationship with the national government and public health policy. The appointment of a greater number of staff members for data entry, and providing them the training for reporting and efficient coordination could potentially help in inputting the information in a shorter time duration. The use of a standardized and consolidated government-authorized system throughout the entire country, where the data for each individual province could also be fully searchable, is also one of the ways to prevent data discrepancies that can be deployed in such countries. The lack of available data or only available after a delay, due to dependence on manual data operations, was also earlier reported by Callaghan et al. [65].

Our study highlighted all resources and processes involved in the information flow of COVID-19 recording and reporting in Indonesia. It provided a comparison of COVID-19 incidence data for all the Indonesian provinces as compared to the national level data to identify the discrepancies. It also offered techniques for developing a comprehensive COVID-19 surveillance system in Indonesia to control and manage the pandemic. A report from Greece also indicated the need for the collection of comprehensive data and transparency in its communication [66].

The findings of our study also revealed the importance of implementing proper information technologies for the integration of health information systems across Indonesia. The need for interoperability to ensure coordinated response and decision-making at national and local levels was also emphasized by Luengo-Oroz et al. [67,68]. By drawing on the lessons learned from Taiwanese, Singaporean, and Hong Kong success stories [69,70], similar technology and surveillance systems can be utilized for the COVID-19 pandemic management with sufficient strategic planning and vision.

### 4.2. Limitations of This Study

The outdated data for some districts may have resulted in a different duration for counting of reported cases, for example, in the case of Bangka Belitung province. Provinces of West Java and South Kalimantan had no change in terms of positive cases, cured cases, and deaths (0, 0, 0) between the national and provincial levels, it is still not confirmed yet whether these provinces just input the same data or based on their investigation. Future assessment of more accurate data collection and reporting practices across levels and observing data at different time points may be warranted.

## 5. Conclusions

In Indonesia, our research found considerable differences in COVID-19 incidence data at the provincial and national levels. It was discovered that nearly half of the laboratories reported PCR test results to the national database, while the other half only reported test results at the provincial level. It showed the lack of adequate reporting infrastructure and resources causing the data inconsistencies. The disparity in COVID-19 incidence data at the province and national levels, as well as their likely causes, revealed the need to strengthen Indonesia’s health information systems for COVID-19 surveillance. The Ministry of Health makes the information flow of COVID-19 recording and reporting available to the public. However, the public is unaware of the discrepancies in reporting at the provincial and national levels. This disparity also indicated that COVID-19 data in Indonesia had not been fully integrated, implying that COVID-19 distribution in Indonesia could be skewed. COVID-19 patient monitoring must also be strengthened to break the disease’s transmission chain and successfully address the problem in Indonesia.

## Figures and Tables

**Table 1:** Comparison of COVID-19 incidence data at national and provincial levels.

No	Provinces	National Level (KawalCOVID-19 and KawalCorona Websites) [13,14]			Provincial Level				Difference					
		Positive Cases	Cured Cases	Deaths	Positive Cases	Cured Cases	Deaths	References	Positive Cases		Cured Cases		Deaths	
1	DKI Jakarta	610,303	501,083	8991	610,303	501,199	9042	[18]	0	0.00%	116	0.02%	51	0.57%
2	Jawa Barat (West Java)	425,206	340,412	5712	425,206	340,412	5712	[19]	0	0.00%	0	0.00%	0	0.00%
3	Jawa Tengah (Central Java)	276,598	218,424	12,135	276,598	227,539	17,386	[20]	0	0.00%	9115	4.17%	5251	43.27%
4	Jawa Timur (East Java)	184,624	157,489	13,293	184,624	158,640	13,601	[21]	0	0.00%	1151	0.73%	308	2.32%
5	Sulawesi Selatan (South Sulawesi)	66,268	62,404	1004	65,738	62,331	996	[22]	530	0.81%	73	0.12%	8	0.80%
6	Kalimantan Timur (East Kalimantan)	81,763	73,072	1957	81,006	72,758	1920	[23]	757	0.93%	314	0.43%	37	1.93%
7	Riau	73,726	67,476	1996	73,706	67,457	1995	[24]	20	0.03%	19	0.03%	1	0.05%
8	Sumatera Barat (West Sumatera)	54,187	48,228	1239	54,186	48,343	1243	[25]	1	0.00%	115	0.24%	4	0.32%
9	Banten	60,672	50,271	1447	63,496	54,639	1625	[26]	2824	4.65%	4368	8.69%	178	12.30%
10	Bali	52,828	48,233	1605	52,828	48,239	1605	[27]	0	0.00%	6	0.01%	0	0.00%
11	Sumatera Utara (North Sumatera)	37,425	33,325	1218	37,425	33,323	1218	[28]	0	0.00%	2	0.01%	0	0.00%
12	Yogyakarta	69,470	52,401	1810	68,100	51,601	1778	[29]	1	0.00%	800	1.55%	32	1.80%
13	Kalimantan Selatan (South Kalimantan)	36,832	34,619	1084	36,832	34,619	1084	[30]	0	0.00%	0	0.00%	0	0.00%
14	Papua	21,276	11,687	213	23,818	22,165	469	[31]	2542	11.95%	10,478	89.66%	256	120.19%
15	Sumatera Selatan (South Sumatera)	30,510	26,697	1540	30,152	26,559	1520	[32]	358	1.19%	138	0.52%	20	1.32%
16	Sulawesi Utara (North Sulawesi)	16,921	15,460	562	16,773	15,447	560	[33]	148	0.88%	13	0.08%	2	0.36%
17	Kalimantan Tengah (Central Kalimantan)	27,342	20,903	552	27,271	24,045	740	[34]	71	0.26%	3142	15.03%	188	34.06%
18	Aceh	19,898	15,380	842	19,893	15,335	841	[35]	5	0.03%	45	0.29%	1	0.12%
19	Sulawesi Tenggara (Southeast Sulawesi)	12,188	10,389	249	12,074	10,412	247	[36]	114	0.94%	23	0.22%	2	0.81%
20	Lampung	23,622	19,058	1141	23,302	19,680	1273	[37]	320	1.37%	622	3.26%	132	11.57%
21	Kepulauan Riau (Riau Islands)	29,456	23,751	623	28,848	23,363	626	[38]	608	2.11%	388	1.66%	3	0.48%
22	Nusa Tenggara Barat (West Nusa Tenggara)	13,436	11,426	497	15,027	13,463	618	[39]	1591	11.84%	2037	17.83%	121	24.35%
23	Papua Barat (West Papua)	12,557	9678	192	12,270	9631	191	[40]	287	2.34%	47	0.49%	1	0.52%
24	Maluku	9952	7699	157	9952	7798	159	[41]	0	0.00%	99	1.29%	2	1.27%
25	Kalimantan Utara (North Kalimantan)	13,982	12,271	207	13,827	12,587	210	[42]	155	1.11%	316	2.58%	3	1.45%
26	Sulawesi Tengah (Central Sulawesi)	14,279	12,871	414	14,161	12,856	412	[43]	118	0.83%	15	0.12%	2	0.49%
27	Bengkulu	11,306	9367	240	11,305	9186	234	[44]	1	0.01%	181	1.97%	6	2.56%
28	Gorontalo	6112	5549	186	5997	5571	186	[45]	115	1.92%	22	0.40%	0	0.00%
29	Jambi	13,617	11,884	288	13,511	11,826	284	[46]	106	0.78%	58	0.49%	4	1.41%
30	Kalimantan Barat (West Kalimantan)	16,227	14,090	411	14,669	13,225	271	[47]	1558	10.62%	865	6.54%	140	51.66%
31	Bangka Belitung	22,613	20,511	354	22,613	20,511	354	[48]	0	0.00%	0	0.00%	0	0.00%
32	Maluku Utara (North Maluku)	6230	4598	139	4414	4117	120	[49]	1816	41.14%	481	11.68%	19	15.83%
33	Nusa Tenggara Timur (East Nusa Tenggara)	21,799	17,114	484	21,642	17,518	514	[50]	157	0.73%	404	2.36%	30	6.20%
34	Sulawesi Barat (West Sulawesi)	6172	5568	126	5933	5505	122	[51]	239	4.03%	63	1.14%	4	3.28%
TOTAL		2,379,397	1,973,388	62,908	2,377,500	2,001,900	69,156		1897	0.08%	28,512	1.44%	6248	9.93%

## Data Availability

Data availability for this article is available online.
